# Health complaints in individual visiting primary health care: population-based national electronic health records of Iran

**DOI:** 10.1186/s12913-022-07880-z

**Published:** 2022-04-14

**Authors:** Farnaz Khatami, Mohammad Shariati, Zahra Abbasi, Taulant Muka, Leila Khedmat, Narges Saleh

**Affiliations:** 1 Department of Community Medicine, School of Medicine, Tehran University of Medical Sciences, Tehran, Iran; 2 Institute of Social and Preventive Medicine (ISPM), University of Bern, Bern, Switzerland; 3 Health Management Research Center, Baqiyatallah University of Medical Sciences, Tehran, Iran; 4 Department of Medical Education, School of Medicine, Tehran University of Medical Sciences, Tehran, Iran

**Keywords:** Clinical complaint, Primary care, Educational needs assessment, Medical education, Electronic health record

## Abstract

**Background:**

The mission of medical schools is a sustainable commitment to orient education, research, and services based on the priorities and expectations of society. The most common complaints of patients from comprehensive health service centers (CHSCs) based on the data from electronic health records were assessed in order to determine primary health care (PHC) priorities for the educational planning of medical students in Iran.

**Methods:**

A population-based national study was designed to assess clinical complaints of patients in all age groups who were referred to CHSCs at least once to be visited by physicians. All the data in the census were extracted from electronic health records in PHC system during 2015–2020, classified by the International Classification of Primary Care 2^nd^ edition (ICPC-2e-English), and statistically analyzed. The total number of complaints that were recorded in the system was 17,430,139.

**Results:**

59% of the referring patients were women. The highest number of referrals was related to the age group of 18–59 years (56.9%), while the lowest belonged to the elderly people (13.3%). In all age and sex groups, the first ten complaints of patients with three top priorities in each category included process (follow-up, consultation, and results exam), digestive (toothache and gum complaint, abdominal pain, and diarrhea), respiratory (cough, sore throat, and runny nose), general (fever, pain, and weakness and fatigue), musculoskeletal (back pain, leg complaint, and knee injuries), endocrine and nutritional (weight gain, Feeding problem, and weight loss), cardiovascular (hypertension, palpitations, and Postural hypotension), neurological (headache, dizziness, and paralysis), sexual dysfunction (vaginal complaint, discharge, and irregular menstruation), and dermatological (pruritus, rash, and inflammation) problems.

**Conclusion:**

High priorities in referring to PHC had a key role in assessing the country's health needs. Since this study was in line with the national pattern of complaints and patients' profile, the present findings can be helpful to amend policy-making, educational planning and curricula development in medical schools.

**Supplementary Information:**

The online version contains supplementary material available at 10.1186/s12913-022-07880-z.

## Background

The fundamental role of general practitioners (GPs) in the health system as well as the specialized and sub-specialized training in hospitals has made the determination of common referrals to GPs one of the priorities of educational planning in medical schools [1, 2]. However, recently there have been concerns that GPs in the early years of work are not prepared to meet the expectations of society [3]. Therefore, the social accountability of the curriculum is to direct all medical educations towards training physicians who are able to meet the health needs of the target community. Meanwhile, one of the most important policies of the World Health Organization (WHO) is to educate the medical students to serve the patients and respond to their needs [4]. The contact of medical students with patients potentially provides an opportunity to develop their clinical reasoning, communication skills, and professional attitudes [5]. On the other hand, community-based and outcome-based medical education not only have an effective role in medical professions but also improve the health status of the society [6]. One of the most important progressions in health care of each community is the electronic health record (EHR). The use of a digital version of a person's overall medical history can considerably facilitate the upgrading of health care delivery and management [7]. Also, the exploitation of EHR under medical school leadership can guarantee the increase of students' skills to access the medical history and patients' care [8]. In Iran, the integrated health system (IHS) has been designed and implemented in order to maintain, and update the information of Iranian Patients' EHRs [9]. The initial version of the online system was presented to the Iran Ministry of Health and Medical Education (IMHME) in 2015 [9, 10]. Health policymakers as well as HCPs need epidemiological information from the community, including what happens at the first level of health care (FLHC) for further planning [11]. It plays a key role in providing services in many developed countries [12]. One of the best coding systems for primary care to classify topics by subject matter is the framework of the International Classification of Primary Care 2^nd^ edition (ICPC-2e-English) [13]. This coding system was successfully applied to primary care in different countries such as Australia, Canada, Germany, Netherlands, and Switzerland [13, 14]. It has a biaxial structure with 17 chapters, each divided into seven components including (i) symptoms and complaints, (ii) diagnostic, screening, and preventive measures, (iii) drugs, treatments, and procedures, (iv) clinical lab tests, (v) administrative, (vi) referrals and other reasons for encounter, and (vii) diseases [15]. On the other hand, the International Classification of Diseases, version 10 (ICD-10) system is more for hospital care and does not well explain the common ill-defined reasons in primary care [16, 17]. ICPC-2e can potentially provide the classification possibility of signs and complaints, which is very appropriate to the FLHC so that it allows the multiple coding for repeated visits of a patient with each complaint until the diagnosis and subsequent follow-up [17, 18]. Although outcome-based educational concepts into the field of medical education were earlier introduced, no comprehensive effort has been made so far to determine the expected outcomes of society from trained physicians. Also, there are limited studies concerning the most common clinical complaints of patients referring to primary health care (PHC) in Iran, whereas most studies were qualitative by relying on the opinions of experts. Therefore, the present study is aimed to classify the most common complaints of referring patients to Iran's comprehensive health service centers (CHSCs) recorded in the IHS from 2015–2020. Classifying the clinical complaints and providing community health priorities at the FLHC were conducted to plan educational programs for medical students by developing strategies and policies for the improved implementation and assessment of the program in the future.

## Methods

### Study design and patients

A population-based national electronic health survey at the beginning of 2020 was conducted to assess clinical complaints of patients who at least once referred to GPs in CHSCs located in the whole country of Iran from 2015 to 2020. It is worth mentioning, primary health care (PHC) in Iran has started with a rural community from 1998, and continued to urban communities to the present day with the aim of achieving health for all. In the past decade, PHC was highly organized and modified based on needs of health system and resulted in a significant decrease in health problems [19, 20]. Family physicians as leaders and managers of the health team in PHC are responsible for providing health services, who are usually close to patients’ residences. Health services include preventive care, treating diseases, and referring eligible patients to secondary or tertiary care, without any prejudice to age, sex, and socioeconomic status to the individuals, families, and communities [4]. Since the COVID-19 epidemic officially started in Iran on February 20, 2020 and our study lasted until the end of January 2020, it was not affected by corona. The electronic health record system was started gradually from villages and small towns to large cities and at the time of this study, 88% of Iranians were registered in the system. The rest of the population either did not apply or belonged to two provinces that were registered in another health record system. The total number of complaints that were recorded in the system was 17,430,139. The census method was used to obtain comprehensive statistics from the beginning of patient registration in the IHS. This study was approved by the Ethics Committee of TUMS, Tehran, Iran. Patients' information in the IHS at the time of data registration was electronically entered after receiving patients' verbal and written consent.

### Electronic data capture system and tools

The IHS of IMHME was used to extract the data. The registered detailed information in IHS was obtained concerning the clinical complaints of patients referring to CHSCs throughout the country. The extracted information for each patient was divided into three categories of sex, age, and the list of symptoms and complaints. The IHS documentation was provided to the research team in the form of information without any raw data. For instance, the number of people who had referred with specific complaints in each age group was known and this information was provided to the research team. The registered complaints were as pre-defined codes based on literature review that could be selected by users (GPs) across the CHSCs. At the time of hiring, all GPs undergo a mandatory several-hour training course to become familiar with the EHR. If there is a new revision in the system later, it will be officially announced to the GPs. It is worth mentioning that in IHS, if the patient has come with numerous complaints or requests, the most important one from the patient's point of view is registered as the main complaint and the rest of the problems are included in the problems list. For example, if the patient comes to preventive immunization and also has a headache, the first is recorded as main complaint and the second on the list of problems. The purpose of our study was only to examine the most common complaints and therefore there is no overlap between complaints. There was the option of "free text" for those complaints that did not find a suitable equivalent in the system and typed the complaint. However, the data cleaning was waived due to the breadth of available data and the small amount of "free text" for the available information, so we only received information about registered complaints, and the percentage of complaints registered as "free text" is not announced. In the next step, the classification was performed according to the ICPC-2e coding system and statistical analyzes were performed.

### Data analysis

The ICPC-2e coding method was used to encode and reclassify an extensive list of more than 350 available complaints after discussing with experts and reviewing the literature. Accordingly, the coding and reclassification of complaints registered in the IHS, based on the ICPC-2e-English version, including 726 codes, was done case by case and independently by two experts separately. For coding, if the two systems were different and some of IHS codes did not exactly match the ICPC-2e, their equivalents were equated in the form of “Others” in different chapters. So, eventually 350 codes of IHS were equated to 202 codes in ICPC-2e (Supp, table S1). Also, a third person was asked to judge when there was no agreement between the two people. The aim of using ICPC-2e was facilitating simultaneous and longitudinal comparisons of clinical primary care practice outside the country. The complaints registered in the system were finally presented in terms of descriptive statistics including frequencies with corresponding percentages, stratified by age groups and sex of the patients.

## Results

**Fig. 1:**
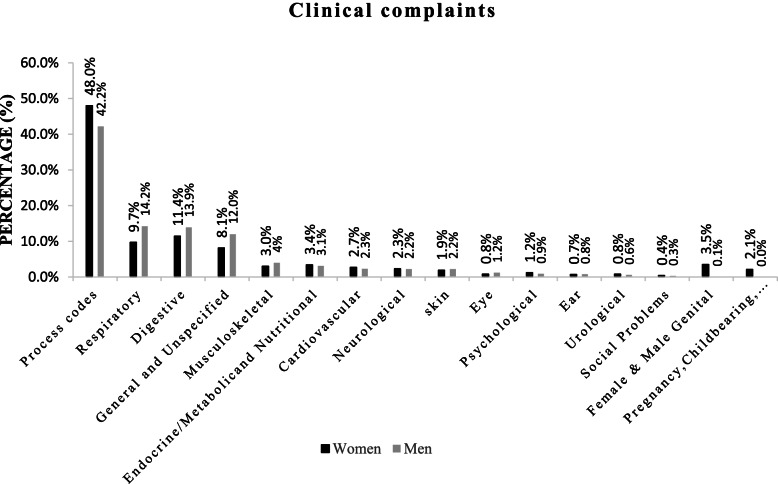
Clinical complaints of men and women referring to Comprehensive health service centers. Complaints are expressed as a percentage, separately for men and women

**Fig. 2:**
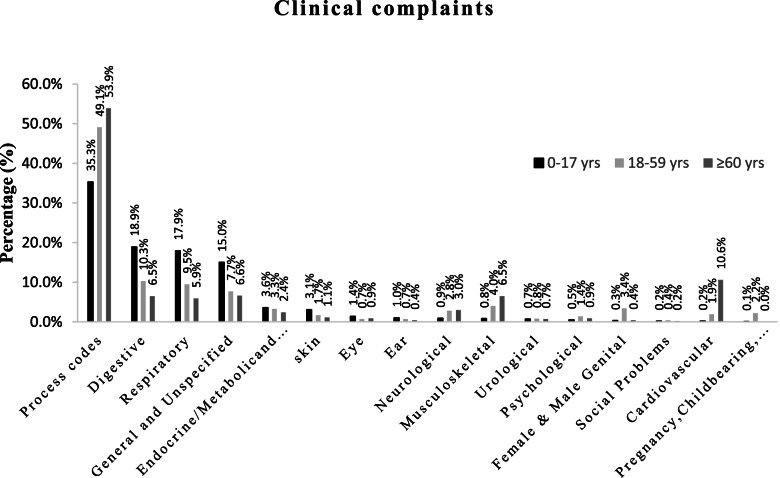
Clinical complaints of Iranians with different age groups, 0–17 years, 18–59 years and ≥ 60 years referring to comprehensive health service centers. Complaints are expressed as a percentage, separately for three age groups

Table 1 shows the description of clinical complaints of Iranian patients referred to CHSCs. 59% of the referring patients were women. The highest number of referrals was related to the age group of 18–59 years (56.9%), while the lowest belonged to the elderly (13.3%). Table 2 mentions priority of main complaints of population including general health issues and complaints, as well as involvements between different organs of the body. Each person had only one of these codes, and patients who had more than one complaint, the main complaint was in this list, but the rest of the complaints or requests were recorded in the list of problems in elsewhere. The most frequent referrals to the GPs present in CHSCs were for issues such as follow-ups and laboratory-clinical tests entitled “process codes (PCs)” (45.7%). Digestive (12.4%), respiratory (11.5%), and general/unspecified (9.7%) complaints were in the next ranks, respectively. Table 3 exhibits the priority of items present in PCs. The most frequent PC-related referrals to CHSCs were for reasons such as follow-up (24.1%), consult with primary care provider (18.9%), Laboratory Test and Results Exam (18.1%). The top priorities in the field of digestive complaints were teeth and gum complaints (45.6%), abdominal pains (19.6%), diarrhea (7.8%), abdominal pain epigastric (6.9%), and vomiting (5%), respectively. Most respiratory complaints included cough (40.8%) and throat symptom (30.6%). The most common general complaints were fever (66.2%), pain (general) (15.4%), weakness and fatigue (6.2%), as well as chills (4.7%), which were ranked in the fourth in the burden of referrals (Table 3). Table 4 shows the musculoskeletal (3.4%), endocrine/metabolic and nutritional (3.3%), cardiovascular (2.5%), neurological (2.3%), genital (2.1%), and skin (2%) complaints as the fifth to tenth priorities of complaints in Iranian patients referred to CHSCs. The most common reasons for referrals due to musculoskeletal complaints included back pain (38.4%), leg and knee complaints (26.9%), as well as muscle pain (6.6%). Nutritional, metabolic, and endocrine disorders with the main reasons such as weight gain (49.4%), feeding problem of adult (28.2%), and weight loss (7.4%) had the sixth rank among the referrals to the CHSCs (Table 4). Although hypertension, palpitation, hypotension, and swollen ankles were the most cardiovascular complaints in patients referred to CHSCs, 92.8% of referrals in this group were due to hypertension only. Headache (61%), dizziness (26.2%), and paralysis or numbness (8.2%) were considered to be the most common reasons for referrals with neurological complaints. According to the ICPC-2e coding system, sexual complaints of men and women were combined. Most sexual complaints are related to vaginal complaints (42.1%) and discharge (22.2%) in women and pain in testis/scrotum (0.9%) in men. Also, pruritus (26.7%), other skin complaints such as skin rash, dry skin, and cracked feet (26.3%) were the most frequent skin complaints among patients. Table 5 reveals patients' clinical complaints in the 11^th^-16^th^ ranks including pregnancy-childbearing-family planning (1.3%), psychological (1.1%), eye (1%), urological (0.8%), ear (0.7%), and social problems (0.4%), respectively. The main corresponding disorders in these complaints were childbirth counseling in both sexes (71.5%), depression, stress, and anxiety (68.4%), itching and discharge from the eyes (71%), frequent urination and foul-smelling urine in both sexes (74.4%), earache (65.7%), and social-psychological complaints (97.8%), respectively. Figure 1 illustrates the clinical complaints of men and women referring to CHSCs. Based on sex stratification analysis, the first four clinical complaint types in men groups were PCs (42.2%), respiratory (14.2%), digestion (13.9%), and general/unspecified complaints (12.0%), whereas the corresponding data in women group were PCs (48.0%), digestive (11.4%), respiratory (9.7%), and general/unspecified (8.1%) complaints, respectively. Figure 2 depicts the prioritization of complaints of different age groups referring to CHSCs. In the age groups of 0–17 and 18–59 years, four main reasons to refer were PCs (35.3 and 49.1%), digestive (18.9 and 10.3%), respiratory (17.9 and 9.5%), and general/unspecified (15.0 and 7.7%) complaints (Fig. 2), in respective order. This order was in an agreement with the main complaints in men groups. It seems that a higher number of men in these age groups referred to CHSCs. In the age group of ≥ 60 years, PCs (53.9%), cardiovascular (10.6%), general/unspecified (6.6%), and digestive (6.5%) complaints were the top main reasons for referring to CHSCs (Fig. 2).

**Table 1:** Baseline description of clinical complaints of Iranian patients referred to CHSCs

Caracteristics	Frequency (n)	Percentage (%)
Total	17,430,139	100
sex		
Female	10,276,495	59
Male	7,153,644	41
age group (years)		
0- 17	5,195,674	29.8
18- 59	9,918,814	56.9
≥ 60	2,315,651	13.3

**Table 2:** The prioritization of the clinical complaints of Iranian patients referred to CHSCs

No	Complaint type	frequency (n)	Percentage (%)
1	Process codes	7,955,109	45.73
2	Digestive	2,158,497	12.38
3	Respiratory	2,011,755	11.54
4	General/Unspecified	1,691,368	9.70
5	Musculoskeletal	588,101	3.37
6	Endocrine/Metabolic, and Nutritional	572,613	3.29
7	Cardiovascular	443,580	2.54
8	Female and Male Genital	368,603	2.11
9	Neurological	397,962	2.28
10	Skin	352,298	2.02
11	Pregnancy, Childbearing, Family Planning	220,777	1.27
12	Psychological	182,538	1.05
13	Eye	167,136	0.96
14	Urological	130,981	0.75
15	Ear	128,181	0.74
16	Social problems	60,640	0.35
17	Blood, Blood Forming, Organs, and Immune Mechanism	0.00	0.00
Total	-	17,430,139	100

**Table 3:** The priorities of clinical complaints in Process codes, digestive, respiratory, and general/unspecified problems among Iranian patients referring to CHSCs

No	**Process codes (1**^**th**^**)**	**Code**	**n (%)**	**Digestive complaint (2**^**th**^**)**	**Code**	**n (%)**	**Respiratory complaints (3**^**th**^**)**	**Code**	**n (%)**	**General/Unspecified complaints (4**^**th**^**)**	**Code**	**n (%)**
1	Follow up	-63	1,915,694 (24.1)	Teeth/Gum complaint	D19	984,223 (45.6)	Cough	R05	820,035 (40.8)	Fever	A03	111,9531 (66.2)
2	Consult with primary Care Provider	-46	1,501,417 (18.9)	Abdominal Pain/Cramps General	D01	423,138 (19.6)	Throat Symptom	R21	616,193 (30.6)	Pain (General)	A01	260,519 (15.4)
3	Laboratory Test and Results Exam	-61	1,436,097 (18.1)	Diarrhea	D11	169,145 (7.8)	Sneezing/nasal congestion	R07	259,679 (12.9)	Weakness and Fatigue	A04	104,892 (6.2)
4	Other Laboratory Test NEC	-38	1,399,871 (16.8)	Abdominal Pain Epigastric	D02	149,346 (6.9)	Respiratory Infection	R83	107,330 (5.3)	Chills	A02	79,759 (4.7)
5	Medicate (Script/Reqst/Renew/Inject)	-50	817,592 (10.3)	Vomiting	D10	107,172 (5.0)	Sputum/phlegm abnormal	R25	59,818 (3.0)	Allergies	A92	60,583 (3.6)
6	Medical Exam/Eval-Complete	-30	335,247 (4.2)	Nausea	D09	91,982 (4.3)	Allergic rhinitis	R97	48,070 (2.4)	Chest Pain	A11	33,295 (2.0)
7	Medical Examination/Health Evaluation/ Pre-op Check	-31	299,943(3.7)	Heartburn	D03	67,146 (3.1)	Shortness of Breath	R02	36,071 (1.8)	Trauma	A80	15,763 (0.9)
8	Local Injection	-55	141,542 (1.8)	Jaundice	D13	55,506 (2.6)	Respiratory Complaint, Others	R29	33,793 (1.7)	Other Bleeding	A10	7,520 (0.4)
9	Other Diagnostic Procedures	-43	130,774 (1.6)	Constipation	D12	43,829 (2.0)	Voice symptom/complaint	R23	7,011 (0.35)	Sweating	A09	3,131 (0.2)
10	Referral to Physician/Specialist/Clinic/Hospital	-67	20,707 (0.3)	Mouth/Tongue/Lip Complaint	D20	23,848 (1.1)	Wheezing	R03	6,305 (0.3)	General Complaint, Other	A29	2,741 (0.2)
11	Preventive Immunizations	-44	14,161 (0.2)	Perianal Itching	D05	13,289 (0.6)	Nose Bleed	R06	6,174 (0.3)	Toxic effect non-medicinal substance	A86	2,576 (0.15)
12	Dress/Press/Compress/Tamponade	-56	1,985 (0.03)	Dyspepsia	D07	10,984 (0.5)	Asthma	R96	4,827 (0.24)	Fainting/syncope	A06	712 (0.04)
13	Diagnostic Radiology/Imaging	-41	79 (0.001)	Parasite in the Stool	D96	4,518 (0.2)	Hypertrophy Tonsils	R90	2,584 (0.13)	Drug Poisoning	A84	262 (0.02)
14	-	-	-	Rectal bleeding	D16	4,240 (0.2)	Foreign Body in the Respiratory System	R87	1,785 (0.1)	Limited Function/Disability NOS	A28	43 (0.003)
15	-	-	-	Melaena	D15	3,333 (0.15)	Breathing Problem, Others	R04	1,301 (0.06)	Feeling Ill	A05	38 (0.002)
16	-	-	-	Anal Fissure	D95	2,504 (0.12)	Whooping Cough	R71	779 (0.04)	Coma	A07	3 (0.0002)
17	-	-	-	Inguinal Hernia	D89	1,915 (0.09)	-	-	-	-	-	-
18	-	-	-	Difficulty Swallowing	D21	1,009 (0.05)	-	-	-	-	-	-
19	-	-	-	Vomiting Blood	D14	707 (0.03)	-	-	-	-	-	-
20	-	-	-	Others (Digestive Complaint)	D29	483 (0.02)	-	-	-	-	-	-
21	-	-	-	Appendicitis	D88	180 (0.01)	-	-	-	-	-	-
**Total**	**-**	**-**	**7,955,109 (100)**	-	-	**2,158,497 (100)**	-	-	**2,011,755 (100)**	-	-	**1,691,368 (100)**

**Table 4:** The frequency (n) and percentage (%) of 5^th^-10^th^ priorities of clinical complaints among Iranian patients referred to CHSCs

No	**Musculoskeletal (5**^**th**^**)**	Code	n	%	**Endocrine/Metabolic and Nutritional (6**^**th**^**)**	Code	n	%	**Cardiovascular (7**^**th**^**)**	Code	n	%
1	Low back pain	L03	225,612	38.4	weight gain	T07	282,868	49.4	Hypertension	K86	411,489	92.8
2	Foot complaint (sprains, pain)	L17	112,812	19.2	Feeding problem of adult	T05	161,478	28.2	Palpitations	K04	15,579	3.5
3	Knee complaint (sprains, pain)	L15	45,164	7.7	weight loss	T08	42,482	7.4	Postural Hypotension	K88	8,266	1.9
4	Muscular pain	L18	38,992	6.6	Loss of appetite	T03	25,562	4.5	Swollen ankles	K07	4,074	0.9
5	Musculoskeletal injuries (orthopedic)	L81	37,550	6.4	Growth delay	T10	20,615	3.6	Cardiovascular complaint, other	K29	1,818	0.4
6	Hand complaint (swelling, pain)	L12	34,479	5.9	Excessive thirst	T01	10,938	1.9	Irregular heartbeat	K05	1,777	0.4
7	Neck complaint (mass, pain, dryness)	L01	29,942	5.1	Feeding problem of infant/child	T04	5,330	0.9	Tightness of heart	K02	577	0.1
8	Shoulder complaint	L08	24,039	4.1	Endocrine complaint, other	T29	5,292	0.9	-	-	-	-
9	Other musculoskeletal complaint (falls, special blows, skeletal pain)	L29	22,475	3.8	overweight	T83	4,831	0.8	-	-	-	-
10	Muscle complaint (strains, contusion)	L19	8,980	1.5	Limited function/disability	T28	4,118	0.7	-	-	-	-
11	Bone fracture	L76	5,160	0.9	Goiter	T81	4,111	0.7	-	-	-	-
12	Ankle complaint	L16	1,603	0.3	Excessive appetite	T02	2,708	0.5	-	-	-	-
13	Osteoarthritis	L91	1,239	0.2	Dehydration	T11	2,280	0.4	-	-	-	-
14	Limited function/disability	L28	54	0.01	-	-	-	-	-	-	-	-
Total		-	**588,101**	100	-	-	**572,613**	100	-	-	**443,580**	100
No	**Neurological (8**^**th**^**)**	Code	n	%	**Female/Male Genital (9**^**th**^**)**	Code	n	%	**Skin (10**^**th**^**)**	Code	n	%
1	Headache	N01	242,847	61.0	Vaginal complaint	X15	155,022	42.1	Pruritus	S02	93,964	26.7
2	Dizziness	N17	104,248	26.2	Vaginal discharge	X14	81,741	22.2	Other skin complaints	S29	92,509	26.3
3	Paralysis/weakness	N18	32,575	8.2	Irregular menstruation	X07	29,024	7.9	Pain/tenderness of skin	S01	38,939	11.1
4	Tingling/burning sensation	N05	9,185	2.3	menstrual complaint	X05-X06	27,082	7.4	Acne	S96	26,055	7.2
5	Convulsions	N07	8,801	2.2	Breast pain female	X18	25,823	7.0	Pediculosis	S73	23,117	6.6
6	Abnormal involuntary movements	N08	306	0.1	Intermenstrual bleeding	X08	17,754	4.8	Animal bites	S13	12,546	3.6
7	-	-	-	-	Breast lump/mass female	X19	12,160	3.3	Skin color change	S08	11,315	3.2
8	-	-	-	-	Menstrual pain	X02	10,574	2.9	Burns	S14	10,878	3.1
9	-	-	-	-	Pain in testis/scrotum	Y02	3,360	0.9	Eczema	S87	10,490	3.0
10	-	-	-	-	Penis complaint, other	X04	1,389	0.4	Hair loss	S23	9,873	2.8
11	-	-	-	-	Genital pain	X01-Y01	1,361	0.4	Rash generalized	S07	8,105	2.3
12	-	-	-	-	Sexual function complaint, male and female	X28-Y08	1,178	0.3	Herpes simplex	S71	5,334	1.5
13	-	-	-	-	Injury male genital	Y80	888	0.2	Lump/swelling localized	S04	3,760	1.1
14	-	-	-	-	Breast complaint, female	X21	536	0.2	Warts and corns	S03-S20	3,515	1.0
15	-	-	-	-	Postcoital bleeding	X13	411	0.1	Skin injury other	S19	1,898	0.5
16	-	-	-	-	Genital herpes	X90	400	0.1	-	-	-	-
Total	-	-	**397,962**	100	-	-	**368,603**	100	-	-	**352,298**	100

**Table 5:** The frequency (n) and percentage (%) of 11^th^-16^th^ priorities of clinical complaints among Iranian patients referred to CHSCs

No	**Pregnancy-Childbearing-Family planning (11**^**th**^**)**	Code	n	%	**Psychological (12**^**th**^**)**	Code	n	%	**Eye (13**^**th**^**)**	Code	n	%
1	Question of pregnancy	W01	157,739	71.5	Feeling depressed	P03	64,729	35.5	Eye symptom/complaint other	F29	58,275	34.9
2	Pregnancy	W78	22,298	10.1	Acute stress reaction	P02	60,042	32.9	Eye discharge	F03	43,721	26.2
3	Contraception other	W14	17,020	7.7	Sleep disturbance	P06	14,757	8.1	Eye pain	F01	30,924	18.5
4	Post-partum symptom/complaint oth	W18	5,693	2.6	Feeling/behaving irritable/angry	P04	12,692	7.0	Glasses symptom/complaint	F17	15,897	9.5
5	Breast/lactation symptom/complaint	W19	4,412	2.0	Psychological symptom/complaint other	P29	9,279	5.1	Red eye	F02	11,019	6.6
6	Infertility/subfertility	W15	4,320	2.0	Tobacco abuse	P17	8,404	4.6	Visual disturbance other	F05	4,103	2.5
7	Post-partum bleeding	W17	2,692	1.2	Feeling anxious/nervous/tense	P01	7,489	4.1	Eye sensation abnormal	F13	1,749	1.1
8	Pregnancy symptom/complaint other	W29	2,429	1.1	Drug abuse	P19	2,239	1.2	Eye appearance abnormal	F15	1,056	0.6
9	Abortion spontaneous	W82	1,660	0.8	Suicide/suicide attempt	P77	973	0.5	Eyelid symptom/complaint	F16	392	0.2
10	Infertility/subfertility male	Y10	1,032	0.5	Memory disturbance	P20	856	0.5	-	-	-	-
11	Contraception intrauterine	W12	730	0.3	Stammering/stuttering/tic	P10	536	0.3	-	-	-	-
12	Pregnancy vomiting/nausea	W05	557	0.3	Limited function/disability	P28	205	0.1	-	-	-	-
13	Pregnancy high risk	W84	195	0.1	Acute alcohol abuse	P16	163	0.1	-	-	-	-
14	-	-	-	-	Child behavior symptom/complaint	P22	83	0.1	-	-	-	-
15	-	-	-	-	Phase of life problem adult	P25	54	0.03	-	-	-	-
16	-	-	-	-	Chronic alcohol abuse	P15	37	0.02	-	-	-	-
**Total**	-	-	**220,777**	100	-	-	**182,538**	100	-	-	**167,136**	100
No	**Urological (14**^**th**^**)**	Code	n	%	**Ear (15**^**th**^**)**	Code	n	%	**Social (16**^**th**^**)**	Code	n	%
1	Urinary frequency/urgency	U02	66,060	50.4	Ear pain/earache	H01	84,223	65.71	Social problem NOS	Z29	59,310	97.8
2	Dysuria/painful urination	U01	31,395	24.0	Acute otitis media//myringitis	H71	20,762	16.20	Relationship problem with partner	Z12	785	1.3
3	Hematuria	U06	10,277	7.9	Plugged feeling ear	H13	15,597	12.17	Assault/harmful event problem	Z25	430	0.7
4	Urine symptom/complaint other	U07	10,127	7.7	Ear discharge	H04	5,982	4.67	Relationship prob. parent/family	Z20	47	0.1
5	Urinary symptom/complaint other	U29	4,377	3.3	Hearing complaint	H02	1,584	1.24	Relationship problem with child	Z16	39	0.1
6	Urination problems other	U05	4,279	3.3	Ear symptom/complaint other	H29	33	0.03	Loss/death of child problem	Z19	29	0.1
7	Incontinence urine	U04	3,821	2.9	-	-	-	-	-	-	-	-
8	Urinary retention	U08	645	0.5	-	-	-	-	-	-	-	-
Total	-	-	**130,981**	100	-	-	**128,181**	100	-	-	**60,640**	100

## Discussion

Awareness of community health priorities is a necessity to achieve the appropriate educational content for medical students. It is worth noting that the consequences of allocating health resources as well as diseases that can face social and economic challenges, play a role in determining the priorities of each community. These priorities are very helpful in the discussion of accountability of medical education which can well reflect the problems and needs of a society. Accordingly, the high prevalence of a disease and its high impact on the patient's life have been accepted as criteria for determining the need to learn diseases in the content of general medical education [21]. In the literature, there are diverse methods in different educational environments to achieve these priorities. At the University of Manchester, O'Neill et al. [22] earlier identified the necessary educational content in two stages using the opinions of GPs, clinical professors, health officials, and medical professionals. In the first stage, a list of clinical situations that GPs should be able to overcome alone, or with guidance, or with the help of a team was recognized. In the second stage, the educational content and the necessary skills and knowledge that GPs should have in dealing with these situations were determined with the help of all doctors and medical staff, as well as colleagues of health. At the University of Sheffield, Newble et al. [22] first prepared a list of clinical signs and symptoms of diseases using textbooks and medical school curricula in order to identify the necessary educational content. The common cases were then selected and prioritized with the opinions of university professors. Tandeter et al. [23] applied the Delphi method with the participation of 40 family physicians and medical instructors to determine the minimum content necessary for general medicine internship. After three Delphi stages, fifteen topics were identified as the most important items to be included in the curriculum. However, our studies were mainly based on the information obtained from Iran's IHS and not according to physicians' opinions.

These findings pave the way for future researches by comparison of the results from the real society with other studies based on opinions of physicians or experts. Finley et al. [24] showed that there was not only a difference between the statements of physicians and patients but also a discrepancy in the rate of PHC referrals between developed and developing countries. Nonetheless, this difference might be useful for the development of PHC guidelines, the allocation of resources, and the design of programs and curricula. On the other hand, it should be noted that this difference may be due to a lack of information in developing countries and therefore does not reflect reality. This fact may also be due to more or less accurate estimates of physicians, as well as their mistakes in diagnosing diseases like mental disorders [24]. There was not enough information about PHC referrals in Iran. This study helps the richness of information in the region and the country. Compared to a similar survey performed in other countries, there are a number of similarities and differences [25].

In the present research, items such as follow-up and request for medical consultation, lab test, digestive complaints such as abdominal pain, and respiratory complaints like cough were identified as priorities to be considered in the educational planning. In a study on referrals to family physicians for four years at the FLSD, the most important diagnoses among patients were hypertension (11.1%), upper respiratory tract infections (URTIs, 11.0%), physical examination (8.6%), diabetes (5.3%), sinusitis (4.8%), bronchitis (4.7%), degenerative joint disease (4.0%), asthma (3.7%), otitis (3.2%), and depression (2.9%), respectively [26]. Another study investigated the time required by physicians to provide preventive services to common chronic diseases at the FLSD. The results revealed that the ten most common chronic diseases included hyperlipidemia, hypertension, depression, asthma, diabetes, arthritis, anxiety, osteoporosis, chronic obstructive pulmonary disease, and coronary artery disease, respectively. Besides, each physician should daily allocate 3.5 working hours to provide preventive services [27]. A practice-based morbidity survey in Birmingham was conducted to examine changes in disease patterns at the FLHC using two disease record systems based on ICD-9 codes. The results showed an increase in gastrointestinal disorders, malignant and benign skin tumors, hypothyroidism, and diabetes. A general decrease in the prevalence of infectious diseases (e.g., conjunctivitis, ear infections, respiratory infections, etc.), acute myocardial infarction, heart failure, and injuries were also observed. This study emphasized the prominent role of GPs in the management of non-communicable diseases [28]. The difference between this study and ours was in the coding system. The goal of our study, was problem-based coding based on ICPC-2e in line with social accountability, but diagnosed disease based on ICD was not considered. However, gastrointestinal and respiratory disorders are among priorities in our results too. To design PHC-specific guidelines in India, 17 primary care centers were asked to report the most common diseases and administered drugs in prescriptions by physicians only with clinical diagnosis. Diseases recorded in order of prevalence included URTIs (45.3%, mainly colds, and acute sore throats), lower respiratory infections (15.9%, mostly bronchitis), parasitic infections (12.6%), anemia (11.4%), dyspepsia as well as ulcer (8.8%), and urinary tract infections (6.1%) [29]. The prevalence rate of URTIs with a high frequency in colds and sore throats was consistent with the present study.

A study in Malaysia compared the incidence and referral patterns of patients according to the top ten priorities of the main complaint and diagnosis in the private and public sectors of primary care using the ICPC-2e coding system over a working week [30]. There was a significant difference in terms of age and sex between the two groups so that the patients in the public sector were older and mostly women. In the public sector, the three main complaints of the patients were respiratory, general, and cardiovascular complaints, respectively. Most patients had chronic and complex diseases such as hypertension and diabetes, as well as pregnancy complaints. In the private sector, the three main complaints of primary care clients were respiratory, general, and digestive complaints, in respective order. It seems that most of the acute patients with respiratory and fever as well as patients with better general conditions had referred to the private sector [30]. When the priorities are compared between these same studies in other countries and our study, as coding system was done on ICPC-2e, there is more similarity including the priority of respiratory, general and digestive complaints in both studies.

In a consistent survey with the present study, Salvi et al. [31] assessed the health profile of all Indian patients throughout the country in all age groups at PHC level. The most common complaints in the general classification were fever (35.5%), headache-body pain (19.5%), loss of appetite (10.2%), and injuries (3.1%), respectively. In our study, fever, pain, and fatigue were also the first three priorities. In the different classification in different organs, the most common reasons for referrals were respiratory symptoms (50.6%), gastrointestinal (25.0%), blood (12.5%), cutaneous (9.0%), and endocrine (6.6%), respectively [31]. In our study, gastrointestinal and respiratory symptoms were in second and third ranks of clinical complaints after PCs.

The demographic composition in the present study is closer to the demographic composition of Iran population in the same period. According to official statistics, 22%, 20% and 40% of the Iranian population were ≤ 20, 21–59 and 60 years old and above, respectively [32]. Interestingly, compared to other groups, there was almost a higher rate of visits among the elder people, which is probably due to the proximity of these centers to the place of residence and the lower cost of services for the elderlies. In addition, women more than men referred to CHSCs. Most of the women referred are under 60 years old. The potential reason for the frequent visits of women is the high prevalence of genital problems, pregnancy, childbearing and family planning in health centers. This statistic is consistent with other studies [33].

The second and third priorities between them were different so that respiratory complaints were more common in men than women. Since these sex groups are different in biology, social roles, and responsibilities are different, due to different risk factors and needs, they will experience various morbidity and mortality. It will be important for policymakers to address these differences for population planning. Studies from different countries showed that health services and health costs were higher in women than men. Here, the burden of diseases should be considered as a significant component for using PHC services between the sexes. A retrospective descriptive study on 79,809 adults referred to PHC using health details in the EHR showed that the use of health services as a result of the higher disease incidence was more in women than men in all age groups. However, there was no significant difference between the two sexes in the use of services and the number of visits after being assigned to the age and burden of infection [34]. A retrospective study in the UK reviewed the non-emergency counseling of GPs and nurses working in the NHS system over a period of 7 years using data recorded in the EHR. Results showed that the number of patients, counseling, and its duration during this period was increased. The highest counseling rate in the age group of 0–4 and ≥ 85 years was observed. Similar to our results, women in all age groups referred more than men [35]. Salvi et al. [31] reported that men in all age groups and geographies referred more compared to women. They explained that this difference may be a result of the sex preference of men in Indian societies. As women and the elder people have the maximum need for health services, fewer reports in this study clearly showed the social inequality in India.

In our study, the first five complaints of Iranian patients were requests, gastrointestinal, respiratory, general, and musculoskeletal, respectively. However, these priorities in clinical complaints are different in other world’s countries due to the discrepancy in health systems, cultural differences, and the burden of disease. For example, pregnancy and family planning, blood/immunity complaints (e.g., HIV), as well as unknown general and neurological causes in South Africa were more prevalent among referrals. However, the same pattern was detected when the 52 most common symptoms/complaints in the Netherlands, Poland, Japan and the United States were compared to the 56 most common causes in South Africa. But, psychological complaints such as depression, anxiety, and sleep disorders beside common complaints in older people (e.g., vision and hearing complaints) were more in these countries compared to the South African. Complaints that appear on the South African list, possibly reflect the burden of HIV/AIDS and tuberculosis (e.g., weight loss, sweating, appetite loss, abnormal sputum, respiratory pain, and dysphagia), and sexually transmitted infections (e.g., genital/pelvic and vaginal pains). In addition, eye-ear infections (e.g., eye pain and discharge, redness of the eyes, and ear discharge), and trauma/injuries were very rare. This fact may indicate different disease loads in these areas [33].

### How to implement these findings to the practice

The profile of health complaints in these visits will reflect social needs in PHC in Iran and will inform the stakeholders in analysis curriculum for determining educational goals and training of medical students and family physicians, as it represents the presentations to which primary care providers must have an evidence based and effective approach. The findings also influence the development of tools and content of educational resources. In the present research, items such as follow-up and request for medical consultation, lab test, digestive complaints such as abdominal pain, and respiratory complaints like cough were identified as priorities to be considered in the educational planning. Curriculum developers in medical schools can plan to enforce these priorities in curriculum. They can encourage Teachers as architects of medical education to build up modules grounding on curricular design principles to fulfill their responsibility of imparting quality education.

### Study strengths and limitations

The most important strengths of this study were the assessment of health needs of the whole country among different age ranges in both sex and also its applicability for the development of educational curricula to train appropriate human resources in order to provide health services. Moreover, the accessibility to information of the most deprived sections of society was provided. In general, there is often a possibility of defects or errors in the actual diagnosis steps because most physicians working in the PHC evaluate the type of patients' disorder/disease based on their medical history and examination without any access to diagnostic methods (required tests or imaging). Therefore, the results of complaints related to the non-use of valid diagnostic methods can lead to adopting a new approach to satisfy patients from the diagnostic and therapeutic process in these healthcare centers. As the information in all seasons over several years was recorded, the collected findings in this study were not affected by seasonal changes in referring patients. Another advantage was the use of the ICPC-2e coding system, which is internationally accepted for the PHC system. Although the conducted literature review was according to the qualitative and interview-based studies, the observational data used in this research were extracted from electronic PHC records. Thus, the research output obtained from our study will have more reliability, complementing previous studies. On the other hand, the main limitation in this study is the underestimation of the real complaints due to no identification of patients' complaints, possible coding errors of the research team, and failure in reporting diagnoses according to the ICPC-2e coding system. Although some of the registered complaints were diagnoses in the coding system, they were not removed from the list due to the value of patients’ information. Besides, all information related to CHSCs with the presence of family physicians was collected from the public sector, while private sector information was not included.

## Conclusions

The present study showed that items such as disease follow-up, consultation request, lab test, and digestive (e.g., abdominal pain) and respiratory (e.g., cough) complaints were identified as priorities to be considered in the educational content. Most patients for the mentioned priorities referred to CHSCs for treatment at the first level of health care. However, medical students spent most of their time in clinical education to learn approaches to refer patient with specified and defined diagnosis, while they did not experience visiting new patients with different signs and symptoms to find diagnose in the FLHC. This study in parallel with the assessment of health needs in Iran's healthcare centers indicates a necessity to partially modify the national pattern of patients' clinical complaints based on their recorded medical profile in the first level of health care. Accordingly, it would contribute to preparing a more comprehensive framework for medical education, educational planning and policy-making, curriculum development, and educational content. Given that the provision of health services in Iran is in line with the needs of society and reflects the socio-economic equality and proportional distribution of government subsidies, a newly developed approach can significantly improve the efficiency of the national programs, guidelines, and policies implemented in PHC for preventive cares.

## Supplementary Information

**Additional file 1:** **Table S1. **Characteristic of equated IHS codeswith ICPC-2e- English codes.

## Data Availability

The data that support the findings of this study are available from The Ministry of Health and Medical Education but restrictions apply to the availability of these data, which were used under license for the current study, and so are not publicly available. Data are, however, available from the authors upon reasonable request and with permission of The Ministry of Health and Medical Education.

## Abbreviations

CHSCs: Comprehensive health service centers
FLHC: First level of health care
EHR: Electronic health record
GPs: General practitioners
HCPs: Health care providers
ICD-10: International Classification of Diseases, version 10
ICPC-2e: International Classification of Primary Care, 2^nd^ edition
IHS: Integrated health system
IMHME: Iran Ministry of Health and Medical Education
PCs: Process codes
PHC: Primary health care
URTIs: Upper respiratory tract infections
