# Supplementary material for: Health complaints in individual visiting primary health care: population-based national electronic health records of Iran
Source: BMC Health Serv Res. 2022 Apr 14;22:502. doi: 10.1186/s12913-022-07880-z (PMC9008379; doi:10.1186/s12913-022-07880-z)
Supplement: Supplementary file 1 — Additional file 1: Table S1. Characteristic of equated IHS codeswith ICPC-2e- English codes. [file 12913_2022_7880_MOESM1_ESM.docx]

**Table S1.** Characteristic of equated IHS codes with ICPC-2e- English codes

| Coding | Numbers |
| --- | --- |
| ICPC-2e-English | 726 |
| IHS codes | 356 |
| Compatible the HIS to ICPC-2e-English | 202 |

Abbreviations: ICPC-2e-English; International Classification of Primary Care, 2^nd^ edition, HIS; Integrated health system.
